# Smart Schools and the Family-School Relationship: Teacher Profiles for the Promotion of Family Involvement

**DOI:** 10.3390/jintelligence11030051

**Published:** 2023-03-08

**Authors:** Begoña Galián, Mª Ángeles Hernández-Prados, José Santiago Álvarez-Muñoz

**Affiliations:** 1 Institute of Educational Sciences, Polytechnic University of Madrid, 28040 Madrid, Spain; b.galian@upm.es; 2 Department of Theory and History of Education, University of Murcia, 30120 Murcia, Spain; mangeles@um.es; 3 Department of Research Methods and Diagnosis in Education, University of Murcia, 30120 Murcia, Spain

**Keywords:** community education, teacher profile, family participation, family school relationship, smart school

## Abstract

Smart schools prioritise work in the educational community, identifying the participation of families as an opportunity, rather than a form of intrusiveness. There are currently a multitude of ways of sharing education with families, ranging from communication to training, with teachers being the driving force in promoting the different roles that families can assume. In this respect, the aim of this cross-sectional, evaluative, non-experimental and quantitative study is to establish the family participation facilitation profiles of 542 teachers working in schools in a multicultural municipality in the autonomous community of the Region of Murcia, in south-eastern Spain. They completed a validated questionnaire with 91 items regarding the different dimensions of family participation, carrying out a cluster analysis to determine the teacher facilitation profiles. The results obtained from the application of the questionnaire show two statistically differentiated teaching profiles. The first of these, with a smaller number of teachers, with fewer years of experience and linked to the pre-primary and secondary stages in public schools, shows less participation in all the modalities considered. In contrast, the profile with the greatest commitment to promoting participation is characterised by having a greater number of teachers, predominantly from state-subsidised centres, that are experienced and linked to the primary stage. In relation to the previous literature, it was possible to confirm the presence of a differentiated profile, finding, on the one hand, teachers interested in the involvement of families and, on the other hand, teachers who do not identify the family–school relationship as a priority. This highlights the need to improve the prior and ongoing training of teachers to raise awareness and sensitise them to the inclusion of families in the educational community.

## 1. Introduction

With the beginning of the 21st century, the educational sphere is entering a new paradigm based on quality as a principle that defines national and international legislation. A model was aimed at improving management, increasing infrastructures and resources, updating the curriculum, and consolidating the role of the teacher as an enabler of quality educational environments and a predictor of the academic success of students as a smart way of addressing quality in schools. Hence, there is a need to evaluate and train for teaching performance in an increasingly complex reality that involves acting “in accordance with their pedagogical competences to guide, guide and evaluate the student’s learning process” (Benavides Gutierrez et al. 2020, p. 48). Despite the leading role that the European regulatory framework has given to pupils, teachers have always been and will always be a crucial element for a healthy functioning education system. A wide variety of teaching competences are recognised, ranging from theoretical, operational, interaction-related and deontological competences (Frabboni 2003) to learning and classroom management, professional commitment, citizenship tutor, and working with the educational community with an inclusive approach to families (López Luján 2018).

Recognising that this is a professional profile in continuous change, open to the creation of new multidisciplinary spaces, focused on offering tools and not just knowledge, is an essential starting point for the pedagogical renewal of schools in order to become smarter (Salmerón Aroca et al. 2023). From this perspective, educational environments are not conceived as hermetic and self-functioning spaces but rather as complementary and necessarily evoking collaboration. Thus, the family, as an exemplary representation of informal education, is potentially valuable in educational centres (formal education) and in associations, neighbourhoods, communities (non-formal education), as Hernández-Prados (2022) has effectively expressed in a recent article. In fact, an intelligent teacher is one who is capable of creating educational spaces that take into consideration the strategic guidelines established by Aranguren-Peraza (2020), among which are the link with other expert agents, especially the family, as they favour school democratisation and the development of flexible mental states that facilitate creativity and learning in the learner, distancing them from unacceptable habits. However, although teachers have the capacity to interact with other people, including families, and despite the appropriateness and intelligence of functioning by establishing links, the reality is that there is a traditional tendency to function in a merely individualistic way (Aguerrondo 2009).

In the same line, at the institutional level, the Organisation for Economic Co-operation and Development [OECD] (2012) considers the involvement of families and communities in the education system, especially in early childhood, as one of the key words for promoting quality in education. In fact, the promotion of family–school collaboration is established as an educational goal in most theoretical-pedagogical approaches. Some perspectives are of an ethical nature, such as the pedagogy of otherness (Hernández-Prados 2014; Vila Merino 2019) or the universal rights of the human being and, therefore, also the rights of children (Castro Alegret et al. 2021). Other pedagogical movements such as the processes of school democratisation (Ochoa 2019), effective and smartschools (Aranguren-Peraza 2020; Mota-Fonseca 2020; Perkins 2019), also place family participation as a relevant element. In other words, if smart schools are to be able to respond to the challenges they face in the most efficient way, successfully employing their strategies and resources, they must establish collaborations with the other educational agents that interact with the child, especially with families. Problems as complex as school failure and dropout are difficult to combat from the field of teaching alone, leaving the families, because as Harji et al. (2016) recognise, a key ingredient of the new smart school curriculum is to increase the participation of parents, because they can play a major role in helping with monitoring the child’s progress, and providing guidance, motivation, and counsel, for example. Without underestimating the ethical perspective that nourishes every educational act that tends towards the improvement and enhancement of the human being, we focus, on this occasion, on the approach to family participation in the framework of smart schools.

The label of smart that is attributed to a school does not depend directly and primarily on the volume of resources at its disposal but rather on it being a school that is able to adapt and understand itself in a context of social and human interaction. This notion, coined by Perkins (1995), stems from the conceptualisation of the school as an organisation open to learning and supportive of the members of the educational community. It is understood as an educational model that dynamises each of the formative, administrative, and communicative processes to promote a social life focusing on the social contexts in which they are located and to promote cultural skills (Flores Hinostroza 2023).

It is far removed from psychometric approaches focused on variables such as IQ or academic results, promoting a notion of intelligence beyond the cognitive, where socio-emotional aspects, among others, have a place (Mota-Fonseca 2020). A smart school is able to understand itself as a scenario of social and human interaction where learning is understood as a product of countless interactions and the well-being of each of its members is promoted (Aranguren-Peraza 2020). 

Taking into consideration the distinction between hard skills (reasoning, interpretation, reflection, abstraction or generalisation) and soft skills (honesty, effective communication, critical thinking, teamwork, motivation, or adaptability) by Heckman and Kautz (2012), “the smart school is the result of a system capable of articulating, in a planned, systematic and consolidated way, cognitive development and life skills, through the implementation of hard skills and soft skills in its classrooms” (Aranguren-Peraza 2022, p. 403).

A smart school is characterised according to Perkins (1995), who distinguished the main characteristics of the smart school: generation knowledge, learnable intelligence, focus on understanding, teaching for mastery and transfer, and others.

It is a humanised institution that promotes thinking, develops the spirit, and nourishes emotions from the following lines of work: promoting happiness, eliminating inadequate habits of the past, building common sense, developing cognitive skills, strengthening interactions, working on social skills, teacher training, research as a practice of action and training, reflection and systematisation of practice, work as a space for recreation and spirituality, the use of ICT, school democratisation, emotional education, and the linking of external agents (Aranguren-Peraza 2020). Based on the latter, smart schools are also characterised by creating a greater link with the family, establishing that the encounter between the family and the educational centres facilitates the understanding of the educational situations of the students, as well as opening, through training workshops, the possibility of reconstruction for those families that need it (Perkins 2019). 

Focusing on the family–school relationship, the benefits of a healthy interaction between both institutions when they reach a consensus for the construction of a common educational project were confirmed (Gomariz et al. 2022; López Luján 2018; Sheridan et al. 2017), especially now that the pandemic has highlighted the need and relevance of strengthening ties in this dyad (Hernández-Prados and Álvarez-Muñoz 2021). This not only evokes improvements for the learner but also for the student body, family, and school, an excellent opportunity to build more democratic and participatory spaces (Ceballos and Saiz 2019). Its relevance was highlighted both in theory, visible in educational policies and legislation, and in practice, increasing good practices that promote the building of bridges of collaboration and communication between family and school (Belmonte et al. 2020). In this way, the conservative view of focusing educational action on students is banished, broadening the focus to families, making teachers aware of the importance of educating and guiding them to exercise positive parenting. In short and taking into consideration all the benefits of the collaboration of families in school activities, it is not wise to waste all this potential. This is recognised by Aranguren-Peraza (2020) when he states that the alliance with families allows for a deeper knowledge of the pupils (needs, aspirations, family coexistence, etc.), as well as helping parents to grow and overcome everyday obstacles. In this sense, schools must implement initiatives to promote the various channels of family participation, and on occasions, the selection of these is carried out without any real knowledge of their potential and foreseeable impact. Proof of this is the scarce presence of this subject in undergraduate studies linked to teaching (Vallespir-Soler and Morey-López 2019), as well as the uncertainty, insecurity and insufficient competence that novice teachers show when facing this world of work for the first time (Monge-Urquijo et al. 2019). In this way, family–school relationships, although containing numerous advantages, are not free of complexities.

When differences between teachers and families are not addressed through dialogue and debate, this can lead to misunderstandings, distancing, or even conflict (Sheridan et al. 2017). This situation leads teachers, instead of perceiving families as collaborators, to identify them as “intruders” (Ceballos and Saiz 2019), creating barriers and building walls that lead to school hermeticism, which is undesirable for the performance of their socialising function. There is no doubt that the lack of recognition by families of the work of teachers has contributed, in part, to the distancing between the two institutions. In this sense, authors such as Guzón-Nestar and González-Alonso (2019) show that the process of dignifying teachers as a key element in the education of children and, consequently, in the construction of the citizenship of the future, begins with the view of families and ends in the social sphere. Therefore, although progress has been made in this field of action, obstacles and difficulties remain. Breaking down these walls implies a greater commitment by teachers to family participation. This requires an effort to seek a rapprochement of positions between both institutions, hence, the relevance of a study that allows us to know the different teaching profiles that exist when referring to collaborative work with families to answer the following research problem: what characterises the teacher as a promoter of family participation at school? Before going into this, it is essential to carry out a brief bibliographical review of the construct: family participation. Specifically, the participation of families and the teacher who facilitates it as essential elements of an intelligent school within the framework of a knowledge society, individualism, and the loss of referents. On this depends, as Aranguren-Peraza (2022) reminds us, the effectiveness of any educational programme towards the improvement or improvement of the human being, especially those centred on emotional and spiritual learning.

### 1.1. Family Participation in Schools

From the theoretical standpoint of smart schools, openness to the community is not something trivial or something that requires an extra effort but rather constitutes a key part of the school’s machinery. The school, apart from its infrastructure and architecture, is the educational encounter between educators and learners, with educators understood in a broad sense, not as a synonym for teachers. It is, therefore incomprehensible, to close the doors of the school to families or to society. Collaboration with families is therefore understood as an important pedagogical pillar for smart schools, which must be able to move beyond purely cognitive knowledge and open up to collaborative experience as an essential framework for community learning that benefits all.

Participation implies activity. It leaves no room for passivity or contemplation. In the words of Navarro Saldaña et al. (2001), the participation of families in school is understood as a duty, giving it an obligatory character, as well as a commitment, since the action is carried out of its own motivation. Duty and commitment are expressions related to the ethical approach to family participation, which prevents parents from looking the other way and denying their share of responsibility in the family–school relationship. However, it also implies a volitional act in which freedom is an unquestionable prerequisite. Values are not imposed, they are shown, so it is the person himself or, in this case, the family who freely decides whether or not to participate. For smart schools, where openness and welcoming are fundamental for the good functioning of coexistence and humanisation, recognising this prevents frustration and burnout syndrome in teachers in the absence of family collaboration but does not justify abandoning their responsibility as facilitators of participation in others (pupils and families). 

Too often we hear that the level of family participation in school is low, at least this is how teachers perceive it (Consejo Escolar del Estado 2014; Munje and Mncube 2018). Furthermore, this lack of family participation is aggravated as children move through the school system, as well as in vulnerable contexts characterised by poverty and immigration (Alonso Carmona 2020; Gálvez 2020). We speak of participation as a construct, being aware of its complexity, associated, on the one hand, with the multitude of school variables (grade, type of school, location, years of teaching experience, academic performance, etc.) and family variables (type of family, parental level of studies, economic situation, etc.) that have an impact on it. Many of them were outlined by Jafarov (2015) after an arduous literature review that allowed up to 22 different factors to be recognised. From the perspective of smart schools, the collaborative relationship with families that humanises educational processes in school contexts should always be maintained, regardless of gender, putting an end to the supposed feminisation of family care (Machancoses 2021); the geographical location of the centre, distinguishing between rural and urban areas (Mangione and Cannella 2021); the level of parental education in favour of those with higher education (Vega Buenaño et al. 2021); or disadvantaged contextual conditions, which, in addition to poverty, family breakdown, lack of empowerment and family work situation, include the attitude of teachers (Lemmer 2007), to cite some variables by way of example.

On the other hand, it is a concept that implies various forms of participation with different levels of intensity or degree of involvement. In this regard, the internationally recognised Epstein (2011) considers six dimensions: parenting, communicating, volunteering, learning at home, decision making, and collaborating with the community. For their part, Sucari et al. (2019), after a systematic review, establish five lines of action for families in school dynamics: as a facilitator of basic conditions for schooling, communication between family and school; as a mediator in academic learning from home, and as a collaborator in the management and activities of the school and contact with the community. According to Vogels (2002), depending on how parents relate to the school, the following profiles of family participation are established: consumers, clients, participants, or partners.

However, the diversity of forms of family participation should not lead to dispersion, nor to the limited offer of some of them by the school. On the contrary, an integrated and complementary view of the different means of participation is required. Thus, “In order to ensure the success of the desired teaching/learning model, smart schools require an effective and efficient management of resources and suitable support processes” (Mangione and Cannella 2021, p. 847). As a result of all this, different parental profiles of participation are determined, overcoming the usual dichotomy that establishes the differentiation between participating and non-participating families, which was reached in the national study of the School Council in Spain (2014). Thus, for the secondary school stage, Hernández-Prados et al. (2019) establish three statistically differentiated profiles: Spanish families with moderate participation and low sense of belonging; families of non-Spanish origin with low participation and moderate sense of belonging; and finally, Spanish families with high participation and high sense of belonging.

The different types of parental participation in schools are a consequence of the prioritisation of needs that accompanies any responsible decision making on how to invest our time. In this respect, teachers should bear in mind that coming to school to be informed or spending the whole afternoon supervising homework is not a highly desirable task (Bailén and Polo 2016). There are many obstacles that make it difficult for families to participate, such as not feeling welcomed or invited by the school (Ceballos and Saiz 2019); or cultural or language difficulties, especially in immigrant families (Gálvez 2020); but the most common are lack of time and difficulties in reconciling work and family life (Belmonte et al. 2020).

The over-accelerated pace of life that society has set plunges today’s citizen into a spiral of obligations and responsibilities. This effect is transferred exponentially to families as a convivial space where different generations converge and was clearly evidenced during the pandemic confinement. Thus, “the unexpected closure of school, teleworking and an imposed conciliation resulted in the oversaturation of family contexts, accompanied by episodes of emotional instability” (Hernández-Prados and Álvarez-Muñoz 2021, p. 28). In this period, families became more vulnerable and unstable, urgently demanding co-responsible educational accompaniment between both institutions. However, this was not always possible or did not reach the desired level. 

Finally, based on the above, the effort made by families to attend and participate in schools should never be underestimated. In the same way, for the sake of educational honesty, schools should carry out an exercise of self-criticism of the meeting experiences they promote and the interest they arouse in families. Only in this way, from the Levinasian approach that forces us to cross over and place ourselves on the other shore (Ortega Ruiz 2010), and from the humanising nature of smart schools, will we be able to carry out the welcoming exercise that families require.

### 1.2. The Teacher as Facilitator of Family Participation

Teachers often complain about low family involvement in schools. This is the reality we have just described. Now, to what extent are teachers willing to open the doors of the school to families so that they really feel invited? Recognising the role of the teacher as a facilitator of family participation is an unquestionable starting point that does not need to be justified. A child’s education does not begin at school but in the family as the first context of reference. Thus, the school acts as a complement to the work initiated by the family, not as a substitute for it. Educational co-responsibility has been claimed by a multitude of studies, to the extent that school functioning and results are limited without the collaboration of families (Jiménez Sánchez 2020; Munje and Mncube 2018; Rivas-Borrell and Ugarte-Artal 2014). 

Unfortunately, at some point in the history of schooling, responsibility for children’s education was carried out unilaterally because of family delegation promoted by the over-acceleration of vital times and the lengthening of working hours (Hernández-Prados and Álvarez-Muñoz 2021). This undesirable situation, which is detrimental to children’s development as it deprives them of the multiple benefits associated with educational co-responsibility between families and teachers, must be reversed. This implies promoting a change not only quantitatively, with a greater presence of families in the school, but also qualitatively, situating the relationship between these agents based on associationism. There is a need for teachers to promote good practices of family participation beyond one-off actions and to incorporate parents as active figures in the teaching–learning processes (Ceballos and Saiz 2019).

Family participation should cease to be anecdotal and should permeate the entire curriculum and the life of the school, becoming a hallmark of the school. However, it seems that collaboration between both institutions is not being sufficiently promoted by teachers (García-Sanz et al. 2020). Similarly, efforts made to foster participation are only a remote possibility in the dark and are hampered by various contextual factors so that the challenges they face are too difficult to overcome (Karıbayeva and Boğar 2014; Munje and Mncube 2018). 

In any case, the school’s own management teams and the parents’ associations see this institution as an excellent opportunity to generate an atmosphere of complicity (Bernad and Llevot 2016). To this end, teachers must stop seeing families as intruders and welcome them as partners. In fact, according to the concept of democratic, smart, and effective schools, it is necessary to start from the knowledge that teachers have of a reality. In this case, teachers must know the role they play in family participation according to the collaboration model established by Gomariz Vicente et al. (2020), which is made up of seven dimensions: communication, participation in activities, feeling of belonging, involvement at home, parents’ associations and School Council, community participation and training.

The actions of teachers when communicating with families are visible from a dual perspective: unidirectional, where messages fluctuate in one direction, or bidirectional, where both parties are active agents of communication, models that are visible to a greater or lesser extent depending on the teaching model (Garreta and Macia 2017). Those teachers who are digitally active show more varied profiles of parental communication since, in addition to using ordinary media, they extend their range of action from the use of new communication channels such as WhatsApp or specific mobile applications (Cascales-Martínez et al. 2020). However, the study by Macià Bordalba (2019) declares the persistence of a teaching activity in which circulars and meetings continue to be more important than technology. There are two ways of acting, in which the innovative contrasts with the traditional and in which profile teachers with a greater or lesser degree of communication with families, conditioned by factors such as the presence of contexts of vulnerability in which they do not have access to technological means (Chávez et al. 2021), the existence of disruptive behaviour among students as a cause of distancing between the two figures (Guzón-Nestar and González-Alonso 2019), or the large number of bureaucracies that teachers have to deal with to the detriment of tasks inherent to tutorial action (González Álvarez 2020). 

Direct participation in the activities organised by the school responds more to the interests of families than to other indirect modalities such as representation in parents’ associations or the School Council (Gomariz et al. 2019). However, teachers’ attitudes differ for various reasons. Some identify the problem in the families themselves, considering that they show little predisposition and interest in participating in the requested activities, with only a small proportion of parents participating (Madrid et al. 2019). Others are not attracted by the idea of parents entering their context of action (Epstein 2011). Both dispositions move away from the smart school’s model, which is committed to the presence of hybrid models in which all members of the community are active agents (Hernández-Prados et al. 2019). However, there are studies that show that in recent years teachers have become more aware of the immersion of families in school activities, designing activities and encouraging families more frequently (Belmonte et al. 2020; Gomariz Vicente et al. 2020; López Luján 2018; Sheridan et al. 2017).

Schools, as public institutions, also involve means of formal participation for parents, underlining the role that parents have in the educational community through the School Council and parents’ associations. However, this is the least desirable line of action for families and the least encouraged by teachers, as they state that they do not carry out any intervention to alleviate this problem (Belmonte et al. 2020; Galián et al. 2018). Promoting the association of families in schools guarantees enormous socio-educational and economic benefits, strengthening the public and administrative image of the school, but in vulnerable contexts marked by poverty, immigration, or cultural mistrust towards education, it is difficult to carry out this task (Murray et al. 2019). Regarding the School Council, in addition to the lack of regulatory knowledge, families argue their non-participation in teacher corporatism, feeling that they are uncomfortable with their lack of knowledge, thus, delaying the functionality of this body (Consejo Escolar del Estado 2014; Rodríguez-Ruiz et al. 2016).

The educational relationships between teachers and parents are not only regulated by what takes place at the educational centre; it is also vital that teachers involve families in the education of children from their daily relationship environment: the home and the community. In this case, previous studies show that teachers tend to make considerable use of families in various tasks outside the school (Gomariz et al. 2019). However, they also show that parents do not correspond much when asked for such collaboration, situations that are a reason for a break with other, more direct forms of communication (Cárcamo and Jarpa-Arriagada 2021). Teachers also highlight the continuous dissemination made by teachers to increase parents’ concern in their children’s education so that they invest time in reducing differences and collaborating in their school tasks instead of delegating this function to external agents (Barrientos Montiel and Arranz de la Fuente 2019).

Likewise, teachers’ difficulties in promoting the participation of families in aspects linked to the community are even more evident, becoming one of the challenges to be solved at school level and from the parental perspective, as both agents do not take care of this dimension of family participation (Fregoso-Borrego et al. 2021). However, there are studies such as that of Barrios et al. (2020) that situate the actions of community participation in collaboration between family and school as evoking a better climate and relationship between both agents, as well as linking the community to the school environment. This unusual form of family participation is claimed by smart schools, as they are flexible, creative, and open to the outside world to promote exchanges (Bazarra and Casanova 2014). According to Aranguren-Peraza (2020), this link with social institutions of cultural relevance (family, companies, town hall, church, neighbourhood community, etc.) represents an opportunity to get to know the students better (families), weave networks of collaboration, humanisation of relationships and spirituality (church), to teach trades (companies), and finally, to create and consolidate creative social spaces for transfer (university).

On the other hand, as a driving force for participation but also because of it, the feeling of belonging to the school is awakened (Gomariz Vicente et al. 2020). From a parental perspective, interest in the school is based on issues directly related to their child, with what transcends the institutional level being irrelevant (Hernández-Prados et al. 2019). Family disinterest is increased by the tendency of teachers to establish differentiated tasks in the educational development of the child instead of joining forces to build a common project (Escalante Rodríguez 2020). Thus, Reparaz-Abaitua and Naval (2014) point to the establishment of personalised treatment as a solution to this problem. In smart schools the student is not the only learner, teachers and families as school stakeholders can and need to learn too (Daly-Smith et al. 2020; Peters et al. 2020). Consequently, it is clear that promoting participation first requires facilitating training for it (Vallespir-Soler and Morey-López 2019). In this sense, teachers should receive training for themselves and promote the participation of families in the training experiences organised by the school and other institutions outside the school. The contingency, ambiguity, and uncertainty in which families find themselves reflects the rupture with the traditional educational model of a patriarchal society and the search for new referents yet to be discovered (Hernández-Prados 2022). Hence, families need to be trained in their educational role, as well as in their collaboration with schools. These dimensions of family participation are not represented or valued in the same way by teachers, establishing a differential development depending on different contextual variables that condition the teaching model in this respect. One of those traditionally studied is gender, as well as the persistent feminisation of family participation, mainly led by mothers (Buxarrais et al. 2019); in the case of teachers, a similar situation occurs when female teachers are identified as those who promote greater contact and rapprochement with families (Cuevas Monzonís et al. 2020). According to the ownership of the centre, more productive relationships are established in those with private or subsidised ownership, as public institutions must overcome situations of difficulty and vulnerability, not so present in other types of centres, that hinder family–school relationships (Cueli and López-Larrosa 2022). Regarding the educational stage (Ceballos and Saiz 2019; Gálvez 2020), most studies determine that as students’ progress through the educational system, parental participation and collaboration in the school decreases (Gomariz et al. 2017). Finally, it is worth mentioning the years of experience; in this case, baggage is a key element that facilitates the establishment of more fluid and fruitful relationships with families, as new teachers still feel insecure in this dimension of being a teacher (Cárcamo-Vásquez and Méndez-Bustos 2019). 

In this way, it is possible to verify that the relationship between family and school is a key element in the scientific field of education. All of them determine differentiated results, alerting of the problems encountered or echoing the improvements in this respect. In most cases, the study is based on the profile of the families and indicates the root of the problems in this agent. However, teachers, as a key player in this relationship, also occupy a place of relevance in the lights and shadows, even more so when the smart school model is mentioned, which underlines the role of teachers as a factor to be taken into consideration. Thus, to improve the family–school relationship, there is a need to know what role teachers play in response to the following research question: Is there a differentiated profile in the role of teachers as facilitators of family participation? This question is represented as an incentive to determine the research objectives of the present investigation.

Thus, there is a broad scientific background in the analysis of the family–school relationship; however, there are few studies that address all the dimensions from a teaching perspective and, even less, their analysis from different socio-demographic variables of interest. Therefore, being aware of the difficulty in competently training teachers for these skills, it is necessary to know their characteristics to be able to plan adapted training. Therefore, the main objective of this research is to obtain teacher profiles according to the degree of facilitation of family participation by teachers. Profiles are understood as natural groupings of teachers that have common characteristics according to a series of selected variables. More specifically, the following specific objectives are proposed:

- To find out whether teachers can be grouped according to how they facilitate family participation.
- To study the characteristics of each of the profiles.
- To compare how teachers of each profile encourage family participation, considering each of the dimensions.
- To analyse the statistically significant differences according to the socio-demographic variables of each of the profiles.

## 2. Methods

The methodology applied in this study is quantitative, non-experimental, and cross-sectional and survey-type. In this sense, the research is focused on describing the extent to which teachers encourage family participation at a specific time, allowing “the identification of individuals with a condition or factor” (Rodrígez and Mendivelso 2018, p. 142).

### 2.1. Sample

The research is contextualised in a rural municipality of Murcia (Spain), Torre Pacheco, where the primary sector and, more specifically, agriculture predominates. In this environment, there is a high percentage of Latin and Arab immigration. The participating population are the teachers of the 14 pre-school, primary, and secondary schools in this municipality. 

These are pre-schools, primary and secondary schools that, following the definition of Davidson et al. (2007), could be defined as intelligent schools, which have to manage their sometimes limited resources with the greatest possible ingenuity, efficiency, and intelligence. Some of the problems faced by the schools in the contexts are directly related to the curricular, such as school failure; to the convivial, such as rejection, marginalisation, discrimination and even the problem of ghettoization; and other problems of participation, especially with families (Tapia-Gutiérrez et al. 2011). All the centres invited are aware of the low levels of family participation and how their efforts to work with families do not produce the expected results, as was stated by the management teams when they went to the centre to pass out the questionnaires. Therefore, with the intention of intelligently managing resources in order to eradicate this problem and improve relations with families, this study begins with a descriptive background. In fact, it is ineffective and, therefore, unwise to approach education only from the school level, neglecting relations with other contexts, as the success of the school level depends to a large extent on collaboration with families (Vigo-Arrazola et al. 2016).

The 542 teachers who make up the total population were invited to participate, but a final sample of 225 teachers was obtained. The research was therefore channelled into the so-called volunteer sampling, located within the non-random sampling techniques. (Hernández-Huescar et al. 2012), which reaches a confidence level of 95% assuming a sampling error of 5%. Looking in more detail at the characteristics of the sample, we obtained representation from all educational centres: 2 state-subsidised schools, 7 pre-school and primary schools, 1 pre-school and basic education school, 1 rural grouped school, and 3 secondary schools. In terms of the socio-demographic characteristics of the teachers, the sex of the teachers, the ownership of the centre in which they teach, the educational stage at which they teach and the years of experience they have in this educational institution were assessed. The distribution of the sample is shown in Table 1.

### 2.2. Information Collection Instrument

The Teaching Facilitation of Family Participation in Educational Institutions questionnaire (QFIS-TP) (Gomariz et al. 2022) was used to collect information, applying it in its entirety. Initially, the participants answered the socio-demographic questions indicated in Table 1 and, subsequently, the 91 items of the closed questionnaire distributed in seven dimensions which are: communication, sense of belonging, involvement at home, participation in school activities, participation in the parents’ association and school council, community participation, and training for teachers and families. This questionnaire is answered with five-level Likert-type scales where the lowest rating corresponds to never/not at all/I do not know if they are carried out/totally disagree and the highest rating is always/much/I make it easy for families to get involved in their organisation/totally agree. Although the categorisation is different depending on the dimension, all scales are answered with 5 levels that are evenly distributed. The internal consistency of the questionnaire is excellent, with a high reliability α = .975 (DeVellis 2003). The content validity of this questionnaire was endorsed by 5 experts in university education and 14 members of primary school management teams. Likewise, using the AMOS program “the construct validity of the QFIS-TP was ratified through the calculation of a confirmatory factorial analysis (CFA) using the structural equations model”. The goodness-of-fit indices (CMIN = 3.872; CFI = .872; RMSEA = .056) indicate a good relationship between the theoretical structures and the empirical data obtained (Gomariz et al. 2022, pp. 5–7).

### 2.3. Procedure

This study is part of a R+D+I intervention project, called: Compartimos la educación. Program for the promotion of family participation in schools (EDU2016-77035-R) granted to the Research Group Compartimos Educaciónof having an impact on the project PID2020-113505RB-I00 funded by the Ministry of Science and Innovation/State Research Agency for the design of a web page for continuous teacher training. This project began with a request for collaboration from the Federation of Parents’ Associations of the municipality of Torre Pacheco FAPAmTP) as they detected a low participation of families in the education of their children. After this, meetings were held with the management teams of these centres, proposing an evaluation and intervention proposal. Thus, teachers, through their management team, were asked to complete the questionnaires online via a link that was sent to the e-mail address of each school. The application of the online questionnaire does not include variables or personal data that allow for the identification of participants, so anonymity is respected. Likewise, as mentioned above, the sampling was of volunteers, so voluntariness and informed consent are also respected. After this, the data were analysed and each centre was provided with an individualised report with the results obtained.

### 2.4. Data Analysis

The data were incorporated into a matrix in the SPSS vs. 24 statistical package. To meet this objective, a two-stage cluster analysis was carried out, given that it was based on a large volume of cases and had both categorical and numerical variables (Rubio-Hurtado and Vilà-Baños 2017). In order to ensure the appropriateness of the application of cluster analysis, the following tests were applied (ibiden):

- To check the independence of the variables, Spearman’s correlation coefficient was obtained, in the numerical variables as well as the contingency coefficient for the categorical variables, with most of the correlations being non-significant.
- The Kolmogorov–Smirnov test was applied to check the normal distribution of the population for the numerical variables and the Chi-square test for the multinomial distribution of the categorical variables. In both cases, the distribution was non-normal. However, although the conditions for applying this technique (independence of the variables in the clustering model, normality in the numerical variables, and multinomial distribution in the categorical variables) are not fully met, “internal empirical tests indicate that this procedure is quite robust, even when these conditions are not met” (Ibid., p. 120), as shown in the results.

Once the profiles were obtained, to compare them with each other, since the normality of the participant sample was not obtained, non-parametric statistics were applied, specifically, the Mann–Whitney U test for the numerical variables, and the Chi-square test was used to analyse the statistically significant differences of the categorical variables. All this using a statistical significance level of α = .05. Finally, the magnitude of the effect is calculated using the contingency coefficient, which, according to Cohen (1988), must reach a value of at least .3.

## 3. Results

### 3.1. Clustering of Teachers According to Their Facilitation of Family Engagement

**Figure 1:**
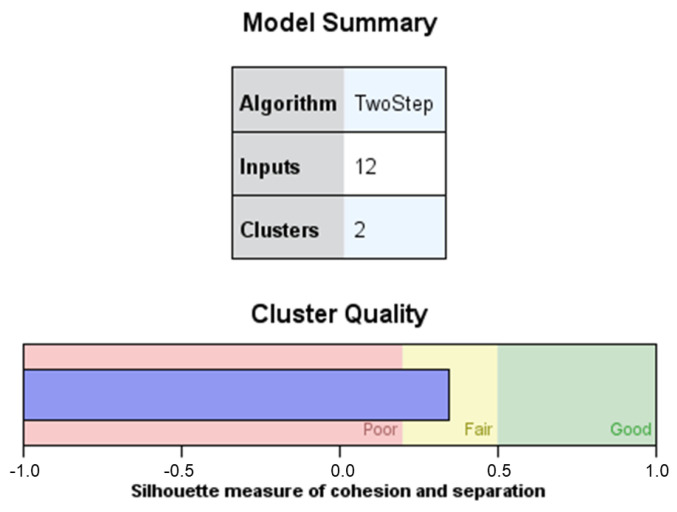
Summary of the model and quality of the clusters.

The summary of the model (Figure 1) shows the configuration of two clusters with the entry of 12 variables: 4 categorical variables (sex of the teacher, educational stage, ownership of the school, and years of teaching experience in the educational institution) and the 8 numerical variables that coincide with the dimensions of the questionnaire, since the dimension involvement in the parents’ associations and in the school council of the school was split into two. Regarding the quality of the clusters, this same figure indicates that the result is in the “Sufficient” zone, which, according to Kaufman and Rousseeuw (1990), is fair but acceptable evidence of the structure of the clusters obtained.

As Table 2 shows, the two clusters obtained are very similar in size. This table shows that profile 2 includes 117 cases, representing 52.2% of the participating teachers; profile 1 is made up of 107 cases, comprising 47.8% of the teachers.

**Figure 2:**
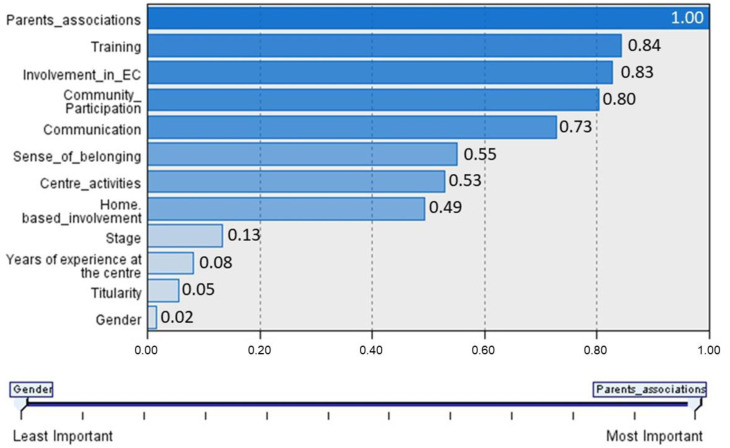
Importance of the predictor in the configuration of the profiles.

The importance of each variable selected to configure the teacher profiles is reflected in Figure 2. As can be seen, the most important predictor for this configuration is the dimension related to the teacher’s facilitation of family involvement in the school’s parents’ associations, followed by the rest of the dimensions included in the questionnaire. The variables stage, experience at the school, ownership of the school, and sex appear as the socio-demographic variables that are important in the configuration of the profiles, although less so than the aforementioned dimensions.

### 3.2. Characteristics of Teaching Profiles

Table 3 shows the frequencies and percentages of each of the categorical variables considered for the formation of the two clusters obtained. Profile 2 is made up of more teachers (57.7%) than profile 1 (42.3%), with an equal number of female teachers in both profiles (50%). Regarding the ownership of the centre, the presence of teachers from public centres is similar in both profiles, highlighting that in profile 2, there is a high presence of teachers from state-subsidised centres (72.4%). About the educational stage, profile 1 is characterised by having more teachers teaching in Pre-school Education (65.6%), in pre-school and primary education at the same time (71.4%), in secondary education and Baccalaureate simultaneously (70.0%), in other stages (66.7%), and in baccalaureate as a whole (100%). On the other hand, profile 2 stands out for a higher presence of teachers in primary Education (68.9%) and secondary education (58.1%). In terms of years of experience at the school, profile 1 is mostly made up of teachers with between 5 and 10 years (59.0%) and between 11 and 20 years of experience at the school (70.0%), in addition to all teachers with more than 30 years (100%) at the educational institution. In contrast, profile 2 is mainly composed of a high percentage of teachers who have been with the school for between 20 and 30 years (85.7%).

Regarding the numerical variables, Table 4 shows that the two profiles of teachers perceive the degree and manner of encouraging family participation in their children’s education differently. Specifically, profile 2 is made up of teachers with a high level of facilitation of family participation, as it stands out for promoting all the dimensions ($\tilde{X}$_Global1_ = 4.11). In contrast, profile 1 consists of teachers with moderate facilitation of family involvement overall ($\tilde{X}$_Global2_ = 3.05), although in this profile, there are dimensions that did not reach the scale standard. These are: encouraging involvement in the parents’ associations ($\tilde{X}$ = 2.50), facilitating involvement in the EC ($\tilde{X}$ = 2.26), and helping with community involvement ($\tilde{X}$ = 2.13).

### 3.3. Comparison between Profiles of the Promotion of Participation Dimensions Families and Socio-Demographic Characteristics

In relation to the numerical variables, Table 5 shows the average range, mean, and standard deviation by dimension for each of the teacher profiles obtained. From these statistics, the statistical significance between dimensions was calculated, considering the cluster of belonging, as well as the effect size, determined by Cohen’s *d* (Cohen 1988).

As a result of applying the Mann–Whitney test, significant differences were found globally and, in all dimensions, depending on the teaching profiles obtained (*p* = .000 in all cases) (Table 5). Likewise, the typical value established by Cohen (*d ≥* .500) was exceeded among all the pairs, i.e., both globally and in all the dimensions, the magnitude of the resulting effect is greater than the standard determined by the author. Regarding the comparison between teaching profiles in terms of categorical variables, the Chi-square test was used to calculate statistical significance and the contingency coefficient to obtain the effect size, which, according to Cohen (1988), must reach at least the value of .300.

As Table 6 indicates, in the case of the sex variable, no statistically significant differences were found between the two profiles (*p* = .323). On the other hand, the contingency coefficient (*r* = .100) did not reach Cohen’s minimum r-value either. About the variables: ownership of the school, educational stage, and the teacher’s experience at the school, significant differences were found between the two profiles obtained (*p* = .020; *p* = .000; *p* = .003, respectively). However, the effect size required by Cohen was only reached for the variables: educational stage (*r* = .337) and experience at the school, by approximation (*r* = .260).

## 4. Discussion and Conclusions

On the road to achieving smart schools, despite the establishment of common teacher training plans and the integration of national programmes to promote family participation, after the results obtained, there are still clearly differentiated views in the educational sphere of familyz–school participation. Thus, regarding the objective of determining the profiles of facilitation of family participation in its different dimensions (communication, participation in activities, feeling of belonging, involvement at home, parents’ associations and school souncil, community participation, and training), two clearly differentiated and dichotomous profiles were identified by means of a cluster analysis. This confirms what was previously stated by Gomariz et al. (2017) about the existence of different teaching styles in terms of how they encourage family participation in the school.

In relation to this, there is too often a tendency to consider participation from a dichotomous perspective; one either participates or one does not participate. This was the case in a national study on family profiles (Consejo Escolar del Estado 2014). However, the existence of various theoretical models that consider multiple dimensions, as discussed in the introduction to this article (Epstein 2011; Gomariz et al. 2022; Sucari et al. 2019), opens a whole range of possibilities in which teachers can promote participation, opting for one or the other. In fact, studies such as Ceballos and Saiz (2019) or Formosinho and Passos (2019) point out that in terms of family participation, teachers comply with the regulations that govern their professional practice, promoting occasional meetings. They hardly leave their comfort zone to engage in other good practices of collaboration that are much more innovative and enriching for the family–school relationship. In fact, according to Bazarra and Casanova (2014), smart schools are knowledge nomads, whereby the entire educational community, but above all the teachers, adopt different roles and acquire new competencies that allow for integrated functioning.

Thus, we find two profiles of teachers when it comes to encouraging family participation. On the one hand, there is the first profile called “not promoting family participation” (profile 1), which is made up of those teachers who are characterised by working in public schools, with a greater volume of new teachers with less than 10 years of experience in the centre and dedicated to the infant, secondary, and baccalaureate stages. This is the profile that brings together the smallest volume of the participating sample.

On the other hand, the second teacher profile, “family engagement facilitator” (profile 2), is characterised by considering families as an equal partner in collaboration (Helgøy and Homme 2017) and is the one with the highest representation in the sample. Thus, most teachers recognise that they do a good job in facilitating family involvement in their schools. These data are contrary to what is expressed by other authors who recognise the lack of invitation and interest on the part of teachers as an obstacle to collaboration between the two institutions (Cheung and Kam 2019; Goodall 2017). Many teachers in this profile are teachers from state-subsidised schools, with many years of experience in the school and mainly dedicated to the primary school stage. The fact that the profile that most encourages participation is made up of most teachers at subsidised schools is related to the change that this type of school has undergone in recent years, since, as Andrés and Giró (2016) indicate, they are more flexible and show a welcoming attitude towards families, which has favoured the creation of a climate of trust.

The years of experience they have at the centre mark, in a statistically significant way, a differentiation in the dynamisation of family participation experiences, in such a way that veterans favour participation more than novices, as shown in both profiles. Thus, teachers with fewer years of experience at the school perceive themselves as less qualified and are more insecure and fearful in their relationship with families (Monge-Urquijo et al. 2019; Vallespir-Soler and Morey-López 2019), which ends up affecting their willingness to promote collaborative encounters with them. In fact, at the beginning of their profession, very few teachers have the knowledge and preparation to implement collaborative experiences that involve families (Epstein 2011). In this sense, teachers require the establishment of continuous favourable experiences to feel capable and skilled in this type of teaching action (Cárcamo-Vásquez and Méndez-Bustos 2019). Moreover, when faced with a new, young, or inexperienced teacher, families show more mistrust, creating barriers between the two figures.

In profile 1, there is a predominance of teaching staff mainly from public schools that encourage less family participation. Profile 2, on the other hand, contains a similar number of teachers from public schools, but also a high percentage of teachers from private or state-subsidised schools. The data suggest that teachers in public schools are less likely to encourage family participation than those in subsidised schools. In this sense, but from the perspective of family participation, Galián et al. (2018) state that teachers in public schools perceive that families participate more than those in private or subsidised schools, so that, although teachers in public schools consider that they encourage less participation, they perceive family participation more frequently than in private schools. In the current reality, the choice of school ownership is a contextual sign, so that private or grant-aided schools are ascribed a high socio-cultural level while public schools have a low socio-cultural level, a contextual difference that has become more evident in recent years (Rizzi 2018). Consequently, teachers in public schools must face situations of vulnerability in which family neglect is one of the most visible features, making it a real challenge for them to contact families and, even more so, to encourage their participation in the dynamics of the school. For this reason, these teachers must educate families in this aspect, while teachers in private or subsidised schools do not have the need to carry out this task of raising awareness and sensitisation regarding their children’s education and the importance of participating (Cueli and López-Larrosa 2022).

When analysing the educational stage of profile 1, there is a higher proportion of teachers in the higher stages. Teachers, and even families, tend to relate the degree of autonomy with greater or lesser involvement of families, a misconception that implies a distancing between the two figures, something that can be seen in research such as that of Parra et al. (2014a) and Hernández-Prados et al. (2019). In pre-school education, it is very common for teachers to invite families to participate in classroom and school dynamics; however, in primary and secondary education, they do not see the need for their participation in the face of a more independent and mature student, banishing the educational possibilities that the immersion of the father figure in school dynamics would have (Gomariz et al. 2015). In profile 2, which stands out for the promotion of family participation, there is a greater presence of teachers in the lower stages, where, as mentioned above, participation is more common (García-Sanz et al. 2016), but teachers should not stop motivating it over the years, as family involvement must continue to be present, albeit through other channels.

Another noteworthy result is related to the gender of teachers (male or female). It is striking that previous studies point to a feminisation of education in both family and school contexts (Machancoses 2021), and it is considered a distinctive variable in parental involvement profiles (Hernández-Prados et al. 2019; Parra et al. 2014b). However, the gender of the participating teachers has not significantly marked the distinction between the two profiles of promoting family participation, or, in other words, it does not influence them when it comes to favouring family participation. What is true is that other authors do consider that there is a difference between how male and female teachers carry out their educational work, with women being more involved and, thus, transmitting gender roles, something that does occur in contexts such as Colombia (Galián 2021).

About the forms of family participation, coinciding with the results obtained, Gomariz et al. (2017) pointed out that of all the forms of family participation considered, the least known and least valued are the formal participation in parents’ associations and school council, training and community participation. This implies that these dimensions require special attention and a greater effort on the part of teachers, both in the first and second profile, to boost family participation in them. This improvement must start with a better knowledge of teachers of these dynamics of participation, as they are also unaware of their functioning and role due to little or no training in this regard (Thompson et al. 2018). Consequently, if this precept is fulfilled, teachers will be able to convey to families the importance of these institutions and what they mean for the school’s educational project; otherwise, without the teacher’s dynamic role, these institutions will persist as spaces for a few and invisible to many.

Coinciding with the findings of other studies (Casado and Galiano 2015; García-Sanz et al. 2016; Gomariz et al. 2019; Paniagua 2013; Parra et al. 2014b), overall in both profiles, participation in the collegiate bodies established by law is the most punished in this respect, although there is a fairly high average difference between them, being more encouraged in profile 2, which is indicative that the promotion of this form of participation is possible depending on the teaching characteristics. In this sense, the low rates of participation may be due to a lack of information, the absence of parents willing to lead this body, and low consideration by the teaching staff, among other causes (Casado and Galiano 2015). More specifically, about the lack of parental involvement in the school council, the lack of competence of families to develop representation in this body is alleged (Gomariz et al. 2019). From a teaching perspective, relevant information is disseminated to encourage this type of participation, but this measure is insufficient (Galián 2021). Finally, the primary school stage is where the greatest normative family participation is observed (Gomariz et al. 2019). Most teachers at this stage are in profile 2. It is, therefore, relevant, in the future, to develop qualitative research that will allow us to identify comparatively the practices of primary school teachers in promoting this form of participation with respect to the rest.

As far as community participation is concerned, it is once again clear that it is the great unknown, at least in the Spanish geographical panorama. Its participation rates are the lowest, coinciding with the data obtained in other studies (Coiduras et al. 2016; Torrego et al. 2019), which state that the few times that contacts between the school and the community take place, they are only occasional. The individualistic vision that prevails in society is also reflected in schools, which are increasingly reluctant to “carry out collective, supportive and cooperative actions” (Giró et al. 2014, p. 86). Breaking this microculture established in the mentality of teachers and families themselves implies alluding to their humanism, as the smart schools trend advocates, and takes advantage of human capital for the common good (Traver et al. 2018), as well as invoking the ethical components of responsibility and commitment that the vulnerability of the other demands (Romero 2014). Therefore, it is necessary to work to ensure that the educational institution is a generator of active citizenship that goes beyond the educational institution itself, positively influencing society (García-Yeste et al. 2013), something that cannot happen without adequate leadership from the management team (Gomariz Vicente et al. 2020).

Finally, despite the emphasis placed on the need for training not only for teachers (Mutton et al. 2018) but also for families (Cano and Casado 2015), we can conclude based on the data obtained in this study that there is no consensus among teachers regarding the role they should play in promoting training qualification experiences for families. In fact, there is clearly a significant difference in averages between profiles in favour of 2, which denotes, on the one hand, a greater awareness among them of the usefulness of this channel for family and child development, widely supported by previous studies (Haisraeli and Fogiel-Bijaoui 2021) that demonstrate the broad human development that is produced by committing to family training. Furthermore, on the other hand, a professional practice committed to the development of its community functions, in addition to its academic functions, places the school close to the society, as contemplated by Aranguren-Peraza (2020) and López Luján (2018) among others.

### 4.1. Implications

Without entering the arduous debate on the professionalism of teachers, as there are different pedagogical positions from which to base their educational practice so that not all proceed in the same way, nor with the same effectiveness, the results obtained in this study have some educational implications, beyond the new lines of research that it promotes, which are worth highlighting. Breaking down walls is undoubtedly one of the major contributions of this research work.

The first wall to break down: the search for blame. The tendency to see something as someone else’s fault is so common in human thinking that when we identify a problem, our brain automatically proceeds to look for possible explanations and culprits to blame. There are still prejudices and stigmas that families have created and that were reinforced on many occasions by the educational institutions themselves, and vice versa, which hinder understanding and nullify the possibilities of collaboration between both contexts (Aranguren-Peraza 2020). This study aims to know not to blame. Educational research cannot be at the service of these preconceptions that contaminate social desirability data, but neither can it ignore them. Smart schools must recognise that improvement is only possible if it is based on knowledge and evaluation of reality. Identifying the messages that stifle family–teacher collaboration, combating them, and building a collaborative environment is the purpose of smart schools. Thus, even admitting the possible bias of desirability, the study shows that not all teachers are comfortable promoting family involvement, even though it is an identifying element of smart schools.

Breaking the overestimation of teacher knowledge: according to Perkins (1995) smart schools should be an informed and dynamic environment that not only promotes reflective learning for students but also provides learning for teachers. In the interaction with families, the same is true; learning must be present for both. Moreover, collaborative tasks should be based on equality. However, sometimes teachers as curriculum and school experts devise and lead collaborative initiatives outside of families. This is unwise, because, as Aranguren-Peraza (2020) and Ramis and Krastina (2010) point out, they arouse little interest in families. We often forget that we can all learn from each other and that everyone has at least one talent to contribute, regardless of whether they are a teacher, a family member, or a student. Third, the uniqueness of participation is another of the walls to be broken down. The model adopted in the study clearly shows the complexity of family participation in schools and the variety of nuances that each of the different ways of participation brings together. The data show that there are fluctuations in the facilitation of the different dimensions of family involvement. Thus, teachers are more comfortable with some forms of participation than others. In this sense, the previous educational and professional background of teachers is represented as a predictor of the predisposition and reality of the family–school relationship (Belmonte et al. 2020; Galián et al. 2018). All this means is that teacher training in family participation cannot be generic or abstract. Rather, it must address different avenues and the peculiarities involved in each of them. There is, in this sense, a manifest need in this work of training in the tools that enable teachers to promote a sense of belonging and community participation in the families of the school. 

Another wall is hierarchical and corporative pressure. Being a teacher in each school implies awakening a certain degree of feeling of belonging but also loyalty to it. However, is this loyalty conditional or unconditional? It is not uncommon for new teachers to become comfortable with the conventional, thus, overriding their renewed vision of education. In this respect, in relation to their work with families, novice teachers nurture their insecurity in this area of work in the face of the negativity of more experienced teachers (Thompson et al. 2018). In this sense, the management team, as the manager and dynamiser of the centre’s identity and culture, plays an important role in promoting family participation. Along these lines, the teachers in charge of the management of the centre must be an example of good practices with families so that they can be extended to the rest of the teaching staff, being a point of reference for the rest of the teachers (Bazarra and Casanova 2014).

Finally, following Senge’s (1992, p. 134) idea that “collaboration makes it possible for the intelligence of the team to be greater than the sum of the intelligence of its members”, we believe that exercising democratic, innovative, and collaborative leadership and promoting teaching tools and strategies that promote collaboration with families is a smart way of connecting the school with the outside world. When it comes to understanding the concept of smart schools, it has traditionally been associated with academic excellence, with the teaching–learning processes within the classroom being the main protagonist. However, a true smart educational institution is one that goes beyond the walls of the school, promoting the relationship with external agents, such as the family, to improve the performance of what takes place in the classroom. In this way, community participation is postulated as a priority line of action in schools in order to truly include families in school dynamics, so that they go from mere spectators to dynamisers.

### 4.2. Limitations and Prospective

The achievement of this research lies in being able to establish groupings of teachers according to how they promote the educational participation of families, as well as making this professional competence, which is not always considered, visible. In this sense, the current study allows for a more detailed assessment and to propose training that is better adapted to the characteristics of the teaching staff. Among its limitations are those related to the quantitative design chosen, the acceptable but improvable volume of participating teachers, as well as the need to listen to other voices (families and students) regarding the strategies used, but also the attitudes expressed, during the process of promoting participation carried out by teachers.

According to Rodrígez and Mendivelso (2018), all research should lead to new questions that allow for the growth of knowledge about what is being researched, such as: What factors lead to a differentiated profile of facilitator of family involvement? Do families have the same perception of teaching profiles? What are the family profiles from the teachers’ perception? Some lines of future work that emerge from this study are: the need to increase, in all training modalities, teacher training with regard to the acquisition of global and specific tools on family involvement; the development, from national and regional administrations, of specific educational programmes in favour of the inclusion of families in formal educational contexts; and, finally, the establishment of a more concrete and practical educational law in this regard.

We conclude this paper by bringing up the results of the European study by Byrne and Paseka (2019), which confirm that irrespective of their social, cultural, or specific context, all parents want the best for their children, including benefits associated with parental involvement. To which we add that the teacher is the professional of reference for making this a reality. The improvement of family participation, and therefore, of educational quality, requires the creation of equitably distributed opportunities that guarantee the probability of participation in conditions that are not always equal for families, i.e., despite difficulties (Murray et al. 2019).

## Figures and Tables

**Table 1:** Frequencies and percentages of the variables that make up the teaching profiles.

Variable	Category	Porcentage	N
Gender	Men	32.4%	73
	Women	67.6%	152
Titularity	Public	87.1%	196
	Concerted	12.9%	29
Stage	Childhood Education	14.2%	32
	Primary Educación	46.2%	104
	Childhood and Primary Education	9.3%	21
	Secondary Education	13.8%	31
	Baccalaureate	0.4%	1
	Secondary Education and baccalaureate	13.3%	30
	Another	14.2%	6
Years of experience at the centre	<5	48.4%	109
	5–10	27.1%	61
	11–20	17.8%	40
	21–30	6.2%	14
	>30	0.4%	1

**Table 2:** Distribution of teaching profiles in the promotion of family participation.

Profile	N	Percentage of Combined
1	107	47.8%
2	117	52.2%
Combined	224	100.0%

**Table 3:** Frequencies and percentages of the variables making up the teaching profiles.

Variable		Profile			
		1		2	
		N	%	N	%
Gender	Men	30	42.3%	41	57.7%
	Women	76	50.0%	76	50.0%
Titularity	Public	99	50.8%	96	49.2%
	Concerted	8	27.6%	21	72.4%
Stage	Childhood Education Primary Educación	21	65.6%	11	34.4%
	Childhood and Primary Education Secondary Education	32	31.1%	71	68.9%
	Baccalaureate	15	71.4%	6	28.6%
	Childhood Education Primary Educación	13	41.9%	18	58.1%
	Childhood and Primary Education Secondary Education	1	100%	0	0%
	Baccalaureate	21	70.0%	9	30.0%
Years of experience at the centre	<5	56	51.9%	52	48.1%
	5–10	36	59.0%	25	41.0%
	11–20	12	70.0%	28	30.0%
	21–30	2	14.3%	12	85.7%
	>30	1	100%	0	0%

**Table 4:** Descriptions of the dimensions of participation by teaching profiles.

Dimension	Profile			
	1		2	
	Average	S.D	Average	S.D
Communication	3.52	.574	4.28	.434
Centre activities	3.08	.781	3.98	.700
Sense of belonging	3.85	.655	4.51	.381
Home-based involvement	4.01	.649	4.62	.358
Involvement in the parents’ associations	2.50	.803	3.99	.802
Involvement in the EC	2.26	.911	3.78	.951
Community Participation	2.13	.771	3.54	.975
Training	3.00	.833	4.17	.563
Global	3.05		4.11	

**Table 5:** Statistical significance and effect size between profiles of the numerical variables.

Dimension	Cluster Number	Average Range	$\tilde{X}$	σ	Mann–Whitney U Test	*p*	*d*
Communication	1	71.29	3.52	.574	1850.000	.000	1.493
	2	150.19	4.28	.434			
Centre activities	1	77.07	3.08	.781	2469.000	.000	1.214
	2	144.90	3.98	.700			
Sense of belonging	1	74.84	3.85	.655	2230.000	.000	1.232
	2	146.94	4.51	.381			
Home-based involvement	1	78.86	4.01	.649	2659.500	.000	1.164
	2	143.27	4.62	.358			
Involvement in the parents’ associations	1	66.00	2.50	.803	1284.000	.000	1.858
	2	155.03	3.99	.802			
Involvement in the EC	1	69.34	2.26	.911	1641.000	.000	1.632
	2	151.97	3.78	.951			
Community Participation	1	70.14	2.13	.771	1727.500	.000	1.604
	2	151.24	3.54	.975			
Training	1	67.05	3.00	.833	1396.500	.000	1.646
	2	154.06	4.17	.563			
Global	1	58.2	3.05	.435	449.000	.000	2.457
	2	162.16	4.11	.436			
Communication	1	71.29	3.52	.574	1850.000	.000	1.493
	2	150.19	4.28	.434			
Centre activities	1	77.07	3.08	.781	2469.000	.000	1.214
	2	144.90	3.98	.700			
Sense of belonging	1	74.84	3.85	.655	2230.000	.000	1.232
	2	146.94	4.51	.381			
Home-based involvement	1	78.86	4.01	.649	2659.500	.000	1.164
	2	143.27	4.62	.358			
Involvement in the parents’ associations	1	66.00	2.50	.803	1284.000	.000	1.858
	2	155.03	3.99	.802			
Involvement in the EC	1	69.34	2.26	.911	1641.000	.000	1.632
	2	151.97	3.78	.951			
Community Participation	1	70.14	2.13	.771	1727.500	.000	1.604
	2	151.24	3.54	.975			
Training	1	67.05	3.00	.833	1396.500	.000	1.646
	2	154.06	4.17	.563			
Global	1	58.2	3.05	.435	449.000	.000	2.457
	2	162.16	4.11	.436			

**Table 6:** Statistical significance and effect size between profiles of the categorical variables.

Categorical Variable	Chi-Square	Statistical Sig. (Chi-Square)	*d*
Gender	2.262	.323	.100
Titularity	5.438	.020	.154
Stage	16.261	.000	.337
Experience at the centre	2.945	.003	.260

## Data Availability

Not applicable.
